# An Effective Chromatography Process for Simultaneous Purification and Separation of Total Lignans and Flavonoids from *Valeriana amurensis*

**DOI:** 10.3390/molecules27238598

**Published:** 2022-12-06

**Authors:** Manli Zhang, Bingyou Yang, Minhui Ye, Jianqing Chen, Yan Liu, Changfu Wang

**Affiliations:** 1 Guangdong Engineering Technology Research Center for Standardized Processing of Chinese Materia Medica, School of Chinese Materia Medica, Guangdong Pharmaceutical University, No. 280 Outside Loop East Road of Higher Education Mega Center, Panyu District, Guangzhou 510006, China; 2 Key Laboratory of Basic and Application Research of Beiyao, Heilongjiang University of Chinese Medicine, Ministry of Education, No. 24 HePing Road, XiangFang District, Harbin 150040, China

**Keywords:** *Valeriana amurensis*, flavonoids, lignans, macroporous resin, polyamide resin

## Abstract

An effective chromatography process was developed and validated for simultaneous purification and separation of total lignans and flavonoids from *Valeriana amurensis*. The total lignans and flavonoids in *Valeriana amurensis* extract were prepurified with macroporous resin column chromatography, and the conditions were optimized as follows: 40 mg/mL *Valeriana amurensis* extract (2.0 g) solution was loaded onto an AB-8 resin column with a diameter-to-height ratio of 1:7, followed by adsorption for 6 h; then, the column was eluted successively with 5 BV water and 10% and 50% ethanol at a flow rate 2 BV/h. The obtained 50% ethanol fraction was further repurified and separated by polyamide resin column chromatography to obtain the total lignans and flavonoids, respectively. The chromatography conditions were optimized as follows: a 50% ethanol fraction (1.0 g) was mixed with 1.0 g polyamide resin and loaded onto a polyamide resin (60–100 mesh) column with a diameter-to-height ratio of 1:3; then, the column was eluted successively with 6 BV water and 40% and 80% ethanol at a flow rate of 4 BV/h. The total lignans and flavonoids were obtained from water and 80% ethanol fraction, respectively. The content and recovery of standard compounds in total lignans and flavonoids were analyzed with HPLC-PDA, and the feasibility of the process was confirmed.

## 1. Introduction

*Valeriana amurensis* Smir. ex Kom (*V. amurensis*) is a member of *Valeriana* belonging to the family *Caprifoliaceae* and is widely distributed in the northeast of China, as well as the Russian Far East and northern Korea. It is particularly abundant in the Greater Khingan Mountains. Roots and rhizomes from *V. amurensis* have been used as the Chinese materia medica of Rhizoma et Radix Valerianae (RERV) [1,2], with pungent and sweet flavor and warm nature, demonstrating mind-calming, qi-regulating and pain-relieving effects [3]. *Chinese Traditional Medicine Resources*, *Northeast Medicinal Plants* and *Heilongjiang Local Medicine* recorded that *V. amurensis* was primarily used for treating nervous system diseases such as insomnia, neurasthenia, anxiety, hysteria and epilepsy [4,5,6]. Compounds isolated or identified from *V. amurensis* include iridoids, lignans, flavonoids, sesquiterpenoids, alkaloids and essential oils [7,8,9,10,11,12]. To date, only an essential oil with a sedative effect has been developed for clinical treatment of insomnia [13]. However, pharmacology studies have revealed that lignans and flavonoids from RERV were also effective components for the treatment of diseases of the nervous system. For instance, lignans of *V. amurensis* are the main components responsible for neuroprotective effects [14], exhibiting significant protective effects against amyloid-beta-induced neurotoxicity in PC12 cells [8,9,15,16]. The flavonoids of RERV have been confirmed to exhibit sedative, hypnotic and anxiolytic effects [17]. Some studies have also proven that flavonoids of RERV comprise another kind of constituent with neuroprotective activity both in vitro and in vivo in Parkinson’s disease [18,19,20]. Therefore, the medicinal value of lignans and flavonoids from *V. amurensis* supports their further development and utilization.

At present, there is no reliable method for simultaneous purification and separation of total lignans and flavonoids from *V. amurensis* or other RERVs. In this study, column chromatographs of macroporous and polyamide resin were used to simultaneously achieve maximal purification and separation of total lignans and flavonoids from *V. amurensis*. The results of this study can effectively promote the development and utilization of lignans and flavonoids of *V. amurensis*, especially the study of their pharmacological effects or synergistic effects on nervous system diseases.

## 2. Results and Discussion

### 2.1. Prepurification Process by Macroporous Resin

#### 2.1.1. Screening of Macroporous Resin

Macroporous resins D101, AB-8 and X-5 were identified as alternatives for effective enrichment of lignans and flavonoids from plant extracts [21,22]. Experimental data on their static adsorption and desorption are shown in Table 1. The adsorption capacity of the resins for total lignans was AB-8 > X-5 > D101, and the adsorption capacity for total flavonoids was AB-8 > D101 > X-5. The desorption capacity of the resins for total lignans was AB-8 > X-5 > D101, and the desorption capacity for total flavonoids was AB-8 > X-5 > D101. Therefore, compared with the X-5 and D101 resins, the AB-8 resin showed more efficient adsorption and desorption to the total lignans and flavonoids in *V. amurensis* extract. As a result, AB-8 resin was selected to purify the total lignans and flavonoids in *V. amurensis* extract.

#### 2.1.2. Adsorption Kinetics Curves

**Figure 1:**
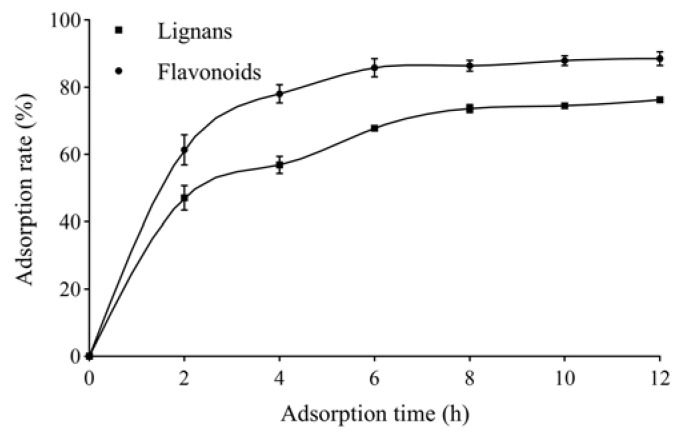
Adsorption kinetics curves for total lignans and flavonoids from *V. amurensis* extract on AB-8 resin.

The adsorption kinetics curves for total lignans and flavonoids on AB-8 resins are shown in Figure 1. The AB-8 resin adsorption process underwent a three-stage change. The adsorption rate showed a linear and rapid increase in the first 2 h and increased slowly from 2 to 6 h, and the resin reached adsorption equilibrium after 6 h.

#### 2.1.3. Sample Loading Concentration

**Figure 2:**
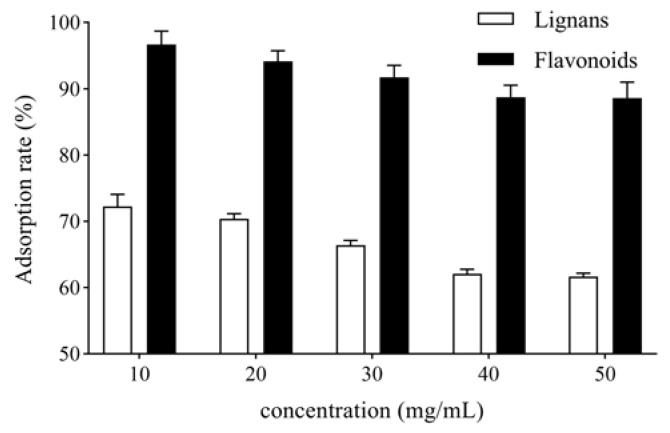
Effect of sample loading concentration of *V. amurensis* extract on the adsorption rate.

*V. amurensis* extract solutions with the concentrations of 10, 20, 30, 40 and 50 mg/mL were prepared by dissolving 0.5, 1.0, 1.5, 2.0 and 2.5 g of *V. amurensis* extract in 50 mL water separately. The solutions were loaded onto AB-8 resin columns. After adsorption for 6 h, the columns were washed with 5 bed volume (BV) water. The unadsorbed solutions and the water-eluted solutions were collected for each column. The adsorption rates of total lignans and flavonoids were determined, as shown in Figure 2. The adsorption rate of total lignans and flavonoids on AB-8 resin decreased with increased sample loading concentration. When the concentration of sample loading was more than 40 mg/mL, the adsorption rate of the AB-8 resin column reached saturation. Therefore, the sample loading concentration was determined to be 40 mg/mL.

#### 2.1.4. Diameter-to-Height Ratio of the Column

The *V. amurensis* extract solution (40 mg/mL) was loaded onto three AB-8 resin columns with diameter-to-height ratios of 1:5, 1:7 and 1:1, allowed to adsorb for 6 h and eluted with 5 BV water to remove impurities before elution with 50% ethanol. The 50% ethanol eluate was collected to determine the purity of total lignans and flavonoids. The corresponding purities of total lignans were 68.14 ± 1.50%, 73.45 ± 1.86% and 59.13 ± 2.49%, and the total flavonoids were 21.82 ± 1.59%, 24.71 ± 0.26% and 18.76 ± 0.68%. Therefore, the optimal diameter-to-height ratio is 1:7.

#### 2.1.5. Desorption Flow Rate

After adsorption for 6 h, the column was eluted with 5 BV water and 50% ethanol. The 50% ethanol eluates were collected at desorption flow rates of 1, 2 and 3 BV/h, and the purity of total lignans and flavonoids was determined. The corresponding purities of total lignans were 54.50 ± 0.60%, 73.45 ± 1.86% and 59.14 ± 2.99%, and total flavonoids were 18.62 ± 0.21%, 24.71 ± 0.26% and 20.81 ± 0.96%. Therefore, the optimal desorption flow rate is 2 BV/h.

#### 2.1.6. Ethanol Concentration

**Figure 3:**
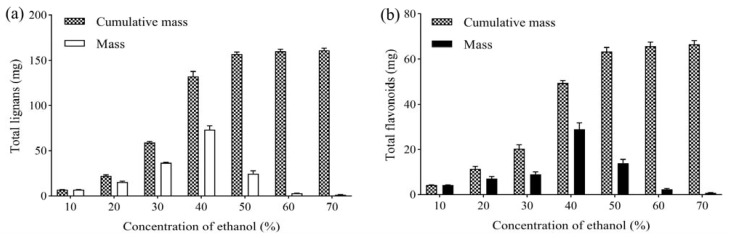
(**a**) Effect of ethanol concentration on mass of total lignans from *V. amurensis* extract; (**b**) effect of ethanol concentration on mass of total flavonoids from *V. amurensis* extract.

After 6 h of adsorption, the column was eluted with 5 BV water. Then, 5 BV 10%, 20%, 30%, 40%, 50% and 60% ethanol was used to elute the column successively, maintaining a flow rate of 2 BV/h. The mass of the obtained total lignans and flavonoids was determined to investigate the effect of eluted ethanol concentration, as shown in Figure 3. Lignans and flavonoids could hardly be detected in 10% ethanol eluate, whereas most flavonoids and lignans were eluted from the column by 50% ethanol. Compared with 50% ethanol, 60% ethanol eluted slightly more lignans and flavonoids from the column. Thus, 10% ethanol was selected to elute impurities, and 50% ethanol was used to elute the total lignans and flavonoids.

#### 2.1.7. Eluent Volume

**Figure 4:**
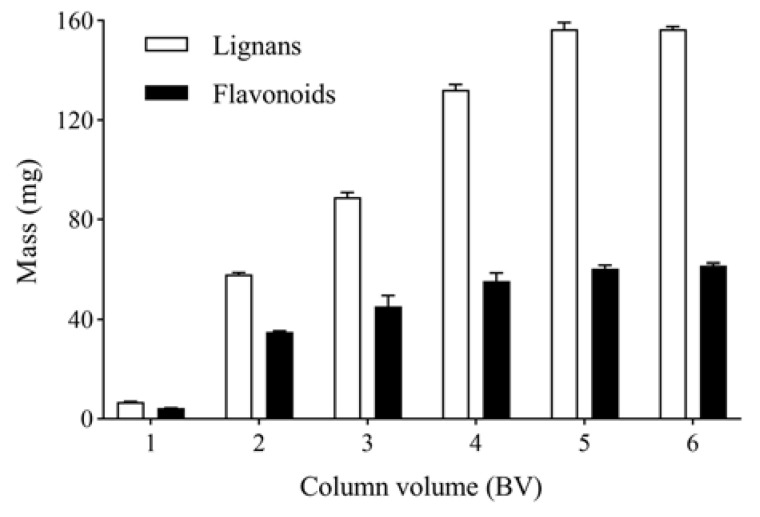
Effect of eluent volume on mass of total lignans and flavonoids from *V. amurensis* extract.

After 6 h of adsorption, 5 BV water and 10% ethanol were used to elute the column. Then, the columns were further eluted with 1, 2, 3, 4, 5 and 6 BV and 50% ethanol at a flow rate of 2 BV/h. The mass of total lignans and flavonoids in 1, 2, 3, 4, 5 and 6 BV and 50% ethanol eluates was detected. The mass of total lignans and flavonoids increased with increased eluent volume. When the eluent volume was 5 BV, the mass of total lignans and flavonoids was stable (Figure 4). Therefore, the optimal eluent volume is 5 BV.

#### 2.1.8. Verification of the Optimal Macroporous Resin Process

Based on the experimental results reported above, the optimal prepurification process for total lignans and flavonoids from *V. amurensis* extract on macroporous resin was determined by chromatography as follows: 2.0 g *V. amurensis* extract was prepared in a 40 mg/mL solution and loaded on an AB-8 resin column with a diameter-to-height ratio of 1:7; after 6 h of adsorption, 5 BV water and 10% and 50% ethanol were used to elute the column successively, maintaining the flow rate at 2 BV/h. The 50% ethanol eluate was collected to obtain P-TLF. The purity of P-TLF was measured to be 66.5 ± 0.8% and 22.8 ± 0.5%, which is 2.7 and 3.8 times that of *V. amurensis* extract (lignans, 24.8%; flavonoids, 6.1%).

### 2.2. Repurification and Separation Process by Polyamide Resin

#### 2.2.1. Screening of Polyamide Resin

The effects of adsorption and desorption properties of three mesh polyamide resins on total lignans and flavonoids were investigated, as shown in Table 2. The 60–100 and 100–200 mesh polyamide resins had the most effective adsorption and desorption rate. Given its efficiency and cost, 60–100 mesh polyamide resin was selected to purify and separate P-TLF.

#### 2.2.2. Sample Loading Mass

Samples 0.5, 1.0 and 2.0 g P-TLF mixed with 1.0 g polyamide resin were loaded onto three polyamide resin columns. The 80% ethanol eluates were collected to detect the purity of total lignans and flavonoids. The results indicate that the corresponding purities of total lignans were 48.98 ± 1.56%, 51.73 ± 0.48% and 46.11 ± 0.81%, and total flavonoids were 20.14 ± 0.74%, 22.33 ± 0.61% and 18.77 ± 0.29%. Therefore, the optimal sample loading mass was determined to be 1.0 g P-TLF.

#### 2.2.3. Diameter-to-Height Ratio of the Column

The 1.0 g P-TLF samples mixed with 1.0 g polyamide resin were loaded onto three polyamide resin columns with diameter-to-height ratios 1:2, 1:3 and 1:4, respectively. The 80% ethanol eluates were collected to detect the purity of total lignans and flavonoids. The results show that the corresponding purities of total lignans were 43.12 ± 2.37%, 51.73 ± 0.48% and 40.74 ± 2.38%, and total flavonoids were 20.45 ± 1.84%, 22.33 ± 0.61% and 19.29 ± 0.81%. Therefore, the optimal diameter-to-height ratio is 1:3.

#### 2.2.4. Desorption Flow Rate

The columns were eluted with 80% ethanol at desorption flow rates of 2, 4 and 6 BV/h. The 80% ethanol eluates were collected to detect the purity of total lignans and flavonoids. The results indicate that the corresponding purities of total lignans were 42.92 ± 0.40%, 51.73 ± 0.48% and 48.81 ± 0.67%, and total flavonoids were 17.35 ± 1.34%, 22.33 ± 0.61% and 20.87 ± 0.98%. Therefore, the optimal desorption flow rate is 4 BV/h.

#### 2.2.5. Eluted Ethanol Concentration

**Figure 5:**
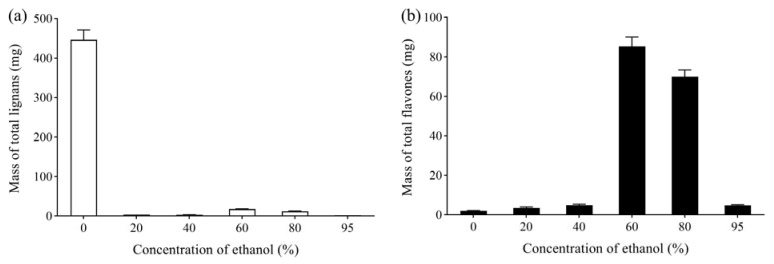
(**a**) Effect of ethanol concentration on the mass of total lignans in P-TLF; (**b**) effect of ethanol concentration on the mass of total flavonoids in P-TLF.

The column was successively eluted with 6 BV water and 20%, 40%, 60%, 80% and 95% ethanol, maintaining the flow rate at 4 BV/h. The mass of total lignans and flavonoids in each eluate is shown in Figure 5. The total lignans were mainly present in the water fraction. Flavonoids could hardly be detected in 40% ethanol eluate, whereas most flavonoids were eluted from the column by 80% ethanol. Compare with 80% ethanol, 95% ethanol eluted only slightly more flavonoids from the column. Thus, water was used to elute the total lignans, and 40% ethanol was selected to elute impurities, whereas 80% ethanol was used to elute the total flavonoids.

#### 2.2.6. Eluent Volume

**Figure 6:**
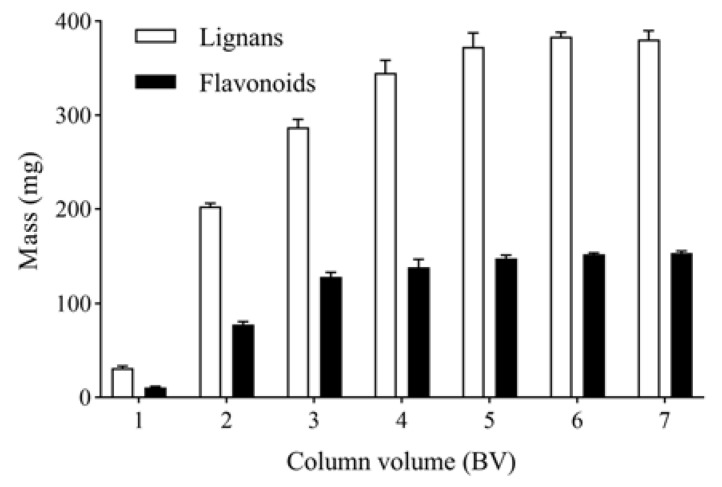
Effect of eluent volume on the mass of total lignans and flavonoids in P-TLF.

The columns were eluted with 1, 2, 3, 4, 5, 6 and 7 BV and water at a flow rate 4 BV/h. The mass of total lignans in water eluates was determined as shown in Figure 6. After eluting with 6 BV and 40% ethanol to remove impurities, the columns were further eluted with 1, 2, 3, 4, 5, 6 and 7 BV and 80% ethanol at a flow rate of 4 BV/h. The mass of total flavonoids in 80% ethanol eluates were determined, as shown in Figure 6. The mass of total lignans and flavonoids increased with increased eluent volume. When the eluent volume was 6 BV, the mass of total lignans and flavonoids was stable. Therefore, the optimal eluent volume is 6 BV.

#### 2.2.7. Verification of the Optimal Polyamide Resin Process

Based on the experiments described above, the optimal repurification and separation process of the P-TLF on polyamide resin was determined as follows: 1.0 g P-TLF mixed with 1.0 g polyamide resin was loaded onto a polyamide resin (60–100 mesh) column with a diameter-to-height ratio of 1:3; then, 6 BV, water, and 40% and 80% ethanol were used to elute the column successively, maintaining the flow rate at 4 BV/h. The purity of total lignans from the water elute was measured to be 75.3 ± 0.8%, which is 1.1 times that of P-TLF (66.5%). The purity of total flavonoids from 80% ethanol elutes was measured to be 68.7 ± 1.5%, which is three times that of P-TLF (22.8%).

### 2.3. HPLC Determination of Lignan and Flavonoid Compounds

**Figure 7:**
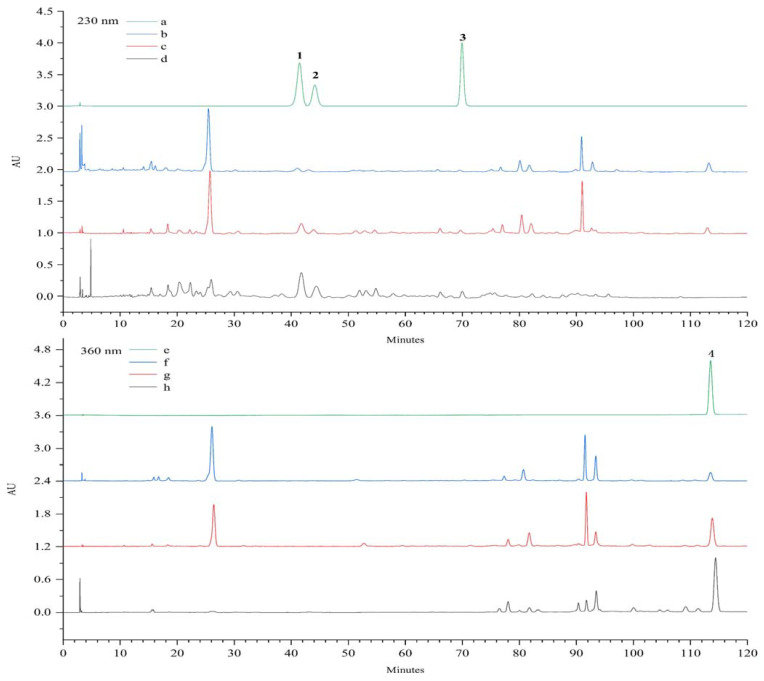
HPLC chromatograms of the standard compounds **1**–**3** (**a**), *V. amurensis* extract (**b**), P-TLF (**c**) and total lignans (**d**) at 230 nm. HPLC chromatograms of the standard compound **4** (**e**), *V. amurensis* extract (**f**), P-TLF (**g**) and total flavonoids (**h**) at 360 nm.

The contents of standard compounds **1**–**4** in *V. amurensis* extract, P-TLF, repurified total lignans and flavonoids were analyzed by HPLC, as shown in Figure 7. Compared with the *V. amurensis* extract, the total lignans and flavonoids were purified and separated significantly after prepurification with macroporous resin and repurification with polyamide resin. The content and recovery of **1**–**4** in *V. amurensis* extract, P-TLF, repurified total lignans and flavonoids are summarized in Table 3. The contents of lignans **1**–**3** and flavonoid 4 were increased significantly, with recovery was up to 83.3%.

## 3. Material and Methods

### 3.1. Plant Materials

The dried roots and rhizomes of *V. amurensis* were provided by Heilongjiang University of Chinese Medicine and were collected from the Greater Khingan Mountains in 2020 and identified by professor Zhenyue Wang. The voucher specimen (No. 20200913) is deposited at the Herbarium of Heilongjiang University of Chinese Medicine, Harbin, China.

### 3.2. Chemicals and Reagents

Standard compounds (purity > 98%) including (+) pinoresinol-4,4’-di-O-β-D-glucopyranoside (**1**), prinsepiol-4-O-β-D-glucoside (**2**), (+) pinoresinol-4-O-β-D-glucopyranoside (**3**) and linarin (**4**) were prepared in our laboratory. AB-8, D101 and X-5 macroporous resins were purchased from Beijing Solarbio Science & Technology Co. Ltd. (Beijing, China). Polyamide resins (30–60, 60–100 and 100–200 mesh) were purchased from Taizhou Luqiao Sijia biochemical plastic factory (Taizhou, China). Aluminum chloride was purchased from Yongda Chemical Reagent Co. Ltd. (Tianjin, China).

### 3.3. Pretreatment of Adsorbents

AB-8, D101 and X-5 macroporous resins were processed according to the method reported in [23]. Polyamide resin (500.0 g) was placed in a round-bottom flask, and 1000 mL 95% ethanol solution (*m/v*, 1:2) was added, followed heating and refluxing for 1.5 h. Then, the solvent was removed by suction filtration. The above operation was repeated twice to obtain clean polyamide resin and dried at 50 °C for further use.

### 3.4. Preparation of V. amurensis Extract

According to a previous report [24], *V. amurensis* extract was prepared as follows: 6.0 kg roots and rhizomes of *V. amurensis* were heated and refluxed with 75% ethanol (*m/v*, 1:8) 3 times for 2 h each time. After removing the solvent, 75% ethanol extract (509.5 g) was obtained. The 75% ethanol extract (500.0 g) was suspended in 8000 mL water, and 8000 mL petroleum ether (60–90°) was added for extraction. The extraction was repeated 8 times until the petroleum ether layer solution was almost colorless. The solvent was removed from the remained aqueous layer solution to obtain 416.7 g *V. amurensis* extract.

### 3.5. Prepurification Process by Macroporous Resin

Samples of 3.0 g of each pretreated D101, AB-8 and X-5 resin were added to a 100 mL conical flask. Then, 50 mL of *V. amurensis* extract aqueous solution (40 mg/mL) was added. The conical flask was put in a constant temperature shaking chamber (25 °C, 120 r/min) to oscillate and adsorb for 24 h. The, the resins were separated from the aqueous solution by filtration, and the concentration of total lignans and flavonoids in the aqueous solution was measured, yielding the adsorption rate (W_a_). The resins were desorbed with 50 mL of 50% ethanol in a constant temperature shaking chamber (25 °C, 120 r/min) for 24 h and filtered to obtain the 50% ethanol solution. Then the concentration of total lignans and flavonoids was measured, yielding the desorption rate (W_d_). W_a_ and W_d_ were calculated using the following formulae:

W_a_ = (C_0_V_0_ − C_1_V_1_)/C_0_V_0_ × 100% (1)

W_d_ = C_2_V_2_/(C_0_V_0_ − C_1_V_1_) × 100% (2)

where C_0_ (mg/mL) is the initial concentration of total lignans or flavonoids in *V. amurensis* extract aqueous solution, V_0_ (mL) is the volume of solution before adsorption, C_1_ (mg/mL) is the concentration of total lignans or flavonoids in *V. amurensis* extract aqueous solution after adsorption, V_1_ (mL) is the volume of solution after adsorption, C_2_ (mg/mL) is the concentration of total lignans or flavonoids after desorption and V_2_ (mL) is the volume of solution after desorption. The optimized resin was determined according to the W_a_ and W_d_ of the resins for total lignans and flavonoids. A resin selection experiment was conducted in parallel three times, and each sample was measured three times. The above experiment was repeated with the optimized resin, and the adsorption rate of total lignans and flavonoids was measured every 2 h 12 h until the adsorption rate reached equilibration, generating the adsorption kinetic curves. *V. amurensis* extract was applied to a chromatography column (15 mm i.d.) filled with the optimized resin. The following indices were used successively: sample loading concentration of *V. amurensis* extract (10, 20, 30, 40 and 50 mg/mL), diameter-to-height ratio of the column (1:5, 1:7 and 1:10), desorption flow rate (1, 2 and 3 BV/h), eluted ethanol concentration (10%, 20%, 30%, 40%, 50%, 60% and 70%) and eluent volume (1, 2, 3, 4, 5 and 6 BV). The indices were optimized by measuring the corresponding parameters, including adsorption rate, mass or purity of total lignans and flavonoids.

### 3.6. Repurification and Separation Process by Polyamide Resin

To obtain the total lignans and flavonoids separately, the prepurified total lignans and flavonoids (P-TLF) from macroporous resin were further purified and separated by polyamide resin column chromatography. According to the methods of static adsorption and desorption described above, 1.0 g of each pretreated polyamide resin (30–60, 60–100 and 100–200 mesh) was placed in 50 mL P-TLF aqueous solution (20 mg/mL) for adsorption. Desorption was carried out with 50 mL of 80% ethanol solution. Next, the resin parameters were determined according to the W_a_ and W_d_ of various mesh polyamide resins for total lignans and flavonoids. P-TLF was applied to a chromatography column (40 mm i.d.) filled with the optimized polyamide resin. The following indices were used successively: sample loading mass of P-TLF (0.5, 1.0 and 2.0 g), diameter-to-height ratio of the column (1:2, 1:3 and 1:4), desorption flow rate (2, 4 and 6 BV/h), eluted ethanol concentration (0%, 40%, 60%, 80% and 95%) and eluent volume (1, 2, 3, 4, 5, 6 and 7 BV). The indices were optimized by measuring the corresponding parameters, including the mass or purity of total lignans and flavonoids.

### 3.7. Quantitative Detection of Lignans and Flavonoids

#### 3.7.1. Conditions of HPLC

A Shim-pack GIST C18-AQ column (4.6 I.D. × 250 mm, 5 μm, Shimadzu, Shanghai, China) was used on an e2695 HPLC couple with a 2998 PDA detector (Waters, Milford, MA, USA) to separate and determine the lignans and flavonoids. After repeated investigation, the chromatography conditions were determined as gradient elution with 0.05% phosphoric acid aqueous solution and methanol at a flow rate 1 mL/min and column temperature of 25 °C. The details are shown in Table 4. Standard compounds **1**–**3** were detected at a wavelength of 230 nm, and compound **4** was detected at a wavelength of 360 nm.

#### 3.7.2. Preparation of Standard Compound and Sample Solutions

A amount of 4.0 mg of each standard compound (**1**–**3**) was dissolved with methanol and diluted into a 25 mL volumetric flask to obtain 0.16 mg/mL mixed stock standard solution. Standard compound (**4**) (2.0 mg) was dissolved with methanol and diluted into a 10 mL volumetric flask to obtain 0.2 mg/mL stock standard solution. All assayed samples including *V. amurensis* extract, P-TLF, total lignans and flavonoids from polyamide resin were dissolved in methanol and passed through a 0.22 μm organic filter membrane.

#### 3.7.3. Method Validation and Quantitative Analysis

HPLC method validation included linearity, precision, repeatability, stability and recoveries. Precision was determined by continuously detecting standard compound solutions. To determine stability, the samples were analyzed at 0, 2, 4, 8, 24 and 48 h. A repeatability test was conducted by analyzing six solutions prepared independently from the same sample. Recovery refers to the assayed mass ratio relative to the theoretical value of a quantitative standard compound added to the sample. The method validations of compounds **1**–**4** were performed according to the methods described above, with shown in Table 5.

### 3.8. Content Determination of Total Lignans and Flavonoids

Compounds **2** and **4** were used as internal standards for the determination of the total lignan and flavonoid contents, respectively. The preparation process of standard compound **2** solutions was the same as the method described above; concentrations of 0.004, 0.008, 0.012, 0.016, 0.020 and 0.024 mg/mL were obtained. Standard compound **4** solutions were prepared according to the method described in [25]. Briefly, 1.0, 1.5, 2.0, 2.5, 3.0 and 4.5 mL of standard solution (0.12 mg/mL) was treated with 4.0 mL of 1% AlCl3-MeOH solution. The reacted solutions were diluted with methanol to 10 mL, and standard solutions with concentrations of 0.012, 0.018, 0.024, 0.030, 0.036 and 0.054 mg/mL were obtained. All samples including *V. amurensis* extract, P-TLF, total lignans and flavonoids from polyamide resin were diluted to an appropriate concentration according to the method described above for a UV spectrophotometry assay. The total lignans and flavonoids was assayed at 230 nm and 386 nm, respectively.

Compound **2** showed good linear correlations (R_2_ = 0.9996) within the range of 0.004–0.024 mg/mL. Satisfactory precision and repeatability were determined, with RSD values of 0.59% and 0.65%, respectively. The sample solution was sufficiently stable within 24 h (RSD = 0.21%). The recovery ability of the method was confirmed, with a recovery range of 97.5% to 101.5% (RSD = 1.64%). Similarly, standard compound **4** was validated in terms of linear correlations (0.012–0.054 mg/mL, R_2_ = 0.9997), precision (RSD = 0.31%), repeatability (RSD = 0.88%), stability (RSD = 0.37%) and recovery from 98.7 to 102.8 (RSD = 1.59%).

## 4. Conclusions

**Figure 8:**
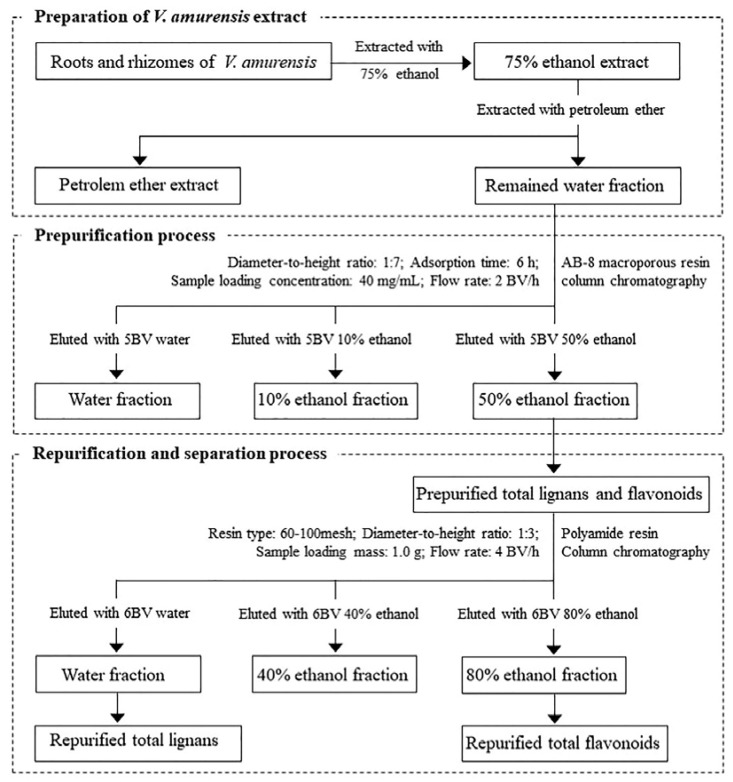
Process optimization on purification and separation of lignans and flavonoids from *V. amurensis*.

We developed and validated an effective chromatography process for simultaneous purification and separation of total lignans and flavonoids from *V. amurensis* with a combination of macroporous and polyamide resins for the first time. Based on the static adsorption and desorption experiments, AB-8 macroporous resin was determined as the optimal adsorbent to prepurify the total lignans and flavonoids from *V. amurensis* extract. Parameters of sample loading concentration, diameter-to-height ratio, desorption flow rate, eluted ethanol concentration and eluent volume were tested and determined, and P-TLF was obtained from the 50% ethanol fraction. To further purify and separate the total lignans and flavonoids, polyamide resin was applied on the P-TLF. The parameters of sample loading mass, diameter-to-height ratio, desorption flow rate, eluted ethanol concentration and eluent volume were optimized, and the optimal process was determined. The total lignans were obtained based on the water fraction, and flavonoids were mainly present in the 80% ethanol fraction. Macroporous resin column chromatography can effectively remove most water-soluble and less polar-liposoluble components in *V. amurensis* extract, the total lignans and flavonoids can be selectively retained. The selective adsorption characteristic of polyamide resin on flavonoids was further utilized to achieve complete purification and separation of total lignans and flavonoids. The preparation of *V. amurensis* extract, as well as prepurification, repurification and separation processes, is shown in Figure 8. Qualitative and quantitative detection and analysis of the standard compounds in the total lignans and flavonoids by HPLC-PDA showed that the recovery for each standard compound was up to 83.3%, and the content was also significantly increased, further confirming the feasibility of the process.

The study results reported herein can effectively promote the development and utilization of flavonoids and lignans of *V. amurensis*, especially the study of their pharmacological effects or synergistic effects on nervous system diseases. Furthermore, herein, we propose a potential process for the large-scale purification and separation of total lignans and flavonoids from *V. amurensis*. The established HPLC method can be applied to quality control.

## Figures and Tables

**Table 1:** Results of static adsorption and desorption experiments with *V. amurensis* extract.

Macroporous Resin	Lignans		Flavonoids	
	W_a_ (%)	W_d_ (%)	W_a_ (%)	W_d_ (%)
D101	58.94 ± 0.18	59.56 ± 0.72	82.78 ± 0.48	71.09 ± 1.56
AB-8	78.90 ± 0.29	84.67 ± 1.11	89.72 ± 1.74	87.31 ± 1.35
X-5	63.19 ± 0.18	71.34 ± 0.35	73.75 ± 0.59	73.11 ± 0.42

Data are described as means ± SD, *n* = 3; W_a_, adsorption rate; W_d_, desorption rate.

**Table 2:** Results of static adsorption and desorption experiments with 50-EF.

Mesh	Lignans		Flavonoids	
	W_a_ (%)	W_d_ (%)	W_a_ (%)	W_d_ (%)
30–60	49.67 ± 0.31	77.48 ± 0.50	89.33 ± 0.45	81.18 ± 0.91
60–100	55.00 ± 0.20	81.82 ± 0.08	90.30 ± 0.34	83.10 ± 1.03
100–200	55.20 ± 0.19	82.14 ± 0.45	90.89 ± 1.23	84.69 ± 1.88

Data are described as means ± SD, *n* = 3; W_a_, adsorption rate; W_d_, desorption rate.

**Table 3:** The content or recovery of standard compounds **1–4** in *V. amurensis* extract, P-TLF, repurified total lignans and flavonoids.

Standard Compounds	Retention Time (min)	*V. amurensis* Extract (μg/mg)	Prepurification		Repurification and Separation		
			P-TLF (μg/mg)	Recovery (%)	Total Lignans (μg/mg)	Total Flavonoids (μg/mg)	Recovery (%)
**1**	114.565	1.14 ± 0.08	4.50 ± 0.17	87.51 ± 3.32	/	16.54 ± 0.67	84.18 ± 3.20
**2**	41.455	3.38 ± 0.11	14.86 ± 0.50	97.37 ± 3.27	22.22 ± 0.81	/	93.55 ± 3.41
**3**	44.124	2.01 ± 0.07	8.86 ± 0.25	97.61 ± 2.72	13.61 ± 0.63	/	96.11 ± 4.46
**4**	69.941	0.73 ± 0.02	2.93 ± 0.11	89.03 ± 3.55	3.93 ± 0.12	/	83.95 ± 2.80

Data are described as means ± SD, *n* = 3.

**Table 4:** The chromatography conditions of HPLC (1 mL/min and 25 °C).

Step	Time (min)	0.05% Phosphoric Acid Aqueous Solution (%)	Methanol (%)
0	0	95	5
1	10	75	25
2	45	75	25
3	50	70	30
4	55	70	30
5	60	65	35
6	70	65	35
7	71	60	40
8	85	60	40
9	86	52	48
10	100	52	48
11	120	45	55

**Table 5:** Method validation for standard compounds **1**–**4**.

Standard Compound	Linear Regression Curve	R^2^	Linear Range (μg)	Precision (RSD)	Repeatability (RSD)	Stability (RSD)	Recovery (%, RSD)
**1**	y = 1,756,083.58x – 44,202.78	0.9998	0.1068–2.128	1.45	1.20	0.76	98.4–102; 1.76
**2**	y = 1,391,241.61x – 74,765.51	0.9999	0.1068–2.128	1.27	1.14	0.85	97.1–101.2; 1.52
**3**	y = 1,627,871.87x – 24,666.6	0.9999	0.1068–2.128	1.08	1.60	1.21	97.3–102.2; 1.87
**4**	y = 2,002,168.43x − 14,490.08	0.9993	0.052–1.072	1.55	1.54	1.14	98.8–102; 1.54

## Data Availability

Data are contained within the article.
